# Bioelectrical Phase Angle in Patients with Breast Cancer: A Systematic Review

**DOI:** 10.3390/cancers14082002

**Published:** 2022-04-15

**Authors:** Delia Morlino, Iolanda Cioffi, Maurizio Marra, Olivia Di Vincenzo, Luca Scalfi, Fabrizio Pasanisi

**Affiliations:** 1 Department of Clinical Medicine and Surgery, Federico II University Hospital, Pansini 5, 80131 Naples, Italy; delia.morlino@unina.it (D.M.); marra@unina.it (M.M.); pasanisi@unina.it (F.P.); 2 Department of Public Health, Federico II University Hospital, Pansini 5, 80131 Naples, Italy; olivia.divincenzo@unina.it (O.D.V.); scalfi@unina.it (L.S.)

**Keywords:** bioimpedance-analysis, muscle strength, chemotherapy, fat mass, survival

## Abstract

**Simple Summary:**

Breast cancer (BC) patients suffer from loss of muscle tissue and fluid alterations during the whole trajectory of the disease. Such alterations might be reflected by phase angle (PhA) measures, but its use in the oncologic setting is still limited. Therefore, the aim of this systematic review was to assess PhA in BC patients, since it has been proven to be a reliable index for predicting nutritional status and survival. Findings reveal that PhA decreases after chemotherapy in BC patients, with high results in women with a better nutritional status, and these changes may persist even after five years. However, PhA remains stable, or can increase in some cases, when patients are supported by targeted lifestyle interventions. Thus, PhA can be useful to identify and monitor changes in body compartments and the nutritional status of BC patients over time.

**Abstract:**

Breast cancer (BC) is the most common cancer diagnosed among women worldwide. Phase angle (PhA), a proxy measure of membrane integrity and function, has gained relevance in clinical practice and it has been suggested to be a prognostic and nutritional indicator. This systematic review aimed to explore PhA and its relationship with nutritional status and survival in BC patients. Four databases (PubMed, EMBASE, Web of Science, and CINAHL) were systematically searched until September 2021 for studies evaluating PhA in BC patients. A total of 16 studies met the inclusion criteria, where 11 were observational studies and 5 were interventional studies. Baseline PhA-value varied from 4.9 to 6.30 degrees, showing a great variability and heterogeneity across the selected studies. Available data suggested that PhA decreased by 5–15% after completing chemotherapy, and those effects might persist in the long term. However, the use of tailored nutritional and/or exercise programs during and after therapy could prevent PhA reduction and body derangement. High PhA values were found in women displaying a better nutritional status, while inconsistent data were found on survival. Therefore, further studies are needed to focus on the clinical relevance of PhA in BC patients, evaluating its association with disease outcomes and survival.

## 1. Introduction

Breast cancer (BC) is the most prevalent cancer and one of the leading causes of death among women worldwide [1,2]. Weight gain might occur very frequently in pre- and post-menopausal women with BC after diagnosis as well as during follow-up, likely due to factors such as the side-effects of chemo- and hormone therapy, early development of menopause, prolonged physical inactivity, and inadequate dietary intakes [3,4,5,6]. The excess of adipose tissue, which can be associated with loss of muscle tissue and body cell mass (BCM) as well as changes in fluid distribution [7], has negative effects on nutritional status and quality of life [8], and has been linked to a high risk of BC recurrence and mortality [9,10].

Bioelectrical impedance analysis (BIA) is a safe, portable, inexpensive, and reproducible bedside method widely used for assessing nutritional status, defining prognosis, and monitoring adult patients suffering from acute and chronic nutrition-related diseases [8,11]. BIA variables [8] are frequently used for the estimation of body composition in the clinical setting, including cancer patients [12,13,14], and for detecting malnutrition and sarcopenia [11]. BIA relies on the electrical properties of human tissues, in terms of impedance, Z; resistance, R; and reactance, X, to estimate fat-free mass (FFM) and total body water (TBW). Thus, the accuracy of such data highly depends on hydration status and the use of appropriate predictive (i.e., age-, sex- and population-specific) equations [8,15]. Over the past years, however, raw BIA variables (mainly phase angle, PhA) have gained great attention since they provide additional information on hydration and FFM composition, in terms of BMC and cell integrity, without requiring specific assumptions, such as constant tissue hydration [11,16,17]. 

BIA-derived PhA, a proxy index of cell membrane integrity and function, is positively correlated with BCM, but inversely related to the ratio of extracellular to intracellular fluid (ECW/ICW) and may be considered as an index of hydration changes [11,16]. A shift of fluid from ICW to ECW with a concomitant increase in the ECW/TBW ratio, may indicate edema or malnutrition [11,17], and is associated with lower PhA [18]. From a practical point of view, PhA has been suggested to be a prognostic, health, functional, and nutritional indicator in various diseases [12,13,14,19,20,21,22,23,24,25]. In patients with different types of cancer, several studies and systematic reviews have shown that a low PhA is associated with an impaired nutritional and functional status [26], decreased quality of life, and increased morbidity and mortality [8,12,26,27]. By contrast, high values of PhA indicated a better nutritional status and a prolonged survival rate [12,14]. 

In light of this background, and considering the lack of previous comprehensive and systematic evaluation of data on BC patients, the aims of this systematic review were: (1) to explore PhA in BC patients in terms of possible differences vs. controls, and variations due to cancer treatments and lifestyle interventions; and (2) to evaluate whether PhA can be used as an index of nutritional status and a predictor of survival. Data on weight and body composition are also reported.

## 2. Materials and Methods

The present systematic review was performed according to the Preferred Reporting Items for Systematic Reviews and Meta-Analysis (PRISMA) Statements [28], the review was not registered. All the steps necessary for the implementation of the systematic review, such as study selection, data extraction, and critical evaluation of the studies, were conducted separately by two authors (D.M. and I.C.). Any disagreement was resolved by discussing with a third review author (L.S.).

### 2.1. Literature Search 

The search strategy was performed until the 22nd of September 2021 using the following electronic databases: PubMed (National Library of Medicine), EMBASE, Cumulative Index to Nursing and Allied Health Literature (CINAHL) and the Cochrane library. The construction of the search strategy was completed using database specific subject headings and keywords. Both medical subject headings (MeSH) and free text search terms were employed. 

The search strategy was conducted using the combination of the following terms “impedance” or “bioelectrical impedance analysis” or “BIA” and phase angle, cancer, breast cancer, body composition, malnutrition, and nutritional status. No filters were applied for study design, language, and publication date. The search strategy was implemented by hand searching the references of all of the included studies and systematic reviews or meta-analyses on the topic.

### 2.2. Eligibility Criteria and Study Selection

To define the eligibility criteria, PICOS (Participants, Interventions, Comparisons, Outcomes, and Study design) criteria were used as follows: “P” (participants) = adult subjects with BC, unrelated to gender and ethnicity; “I” (interventions) = use of BIA for evaluating PhA; “C” (comparisons) = differences of PhA due to patients’ characteristics, treatments, and lifestyle interventions; “O” (outcomes) = PhA measured at 50 kHz; “S” (study design) = all type of studies. 

Articles were selected according to the following characteristics: (1) including subjects aged 18 years or older diagnosed with BC; and (2) performing different types of BIA methods such as single or multifrequency BIA, vector BIA (BIVA), bioelectrical spectroscopy (BIS), or segmental-BIA able to provide PhA values.

Studies were excluded if: (i) participants were aged < 18 years; (ii) data from BIA, collecting from BC patients, could not be extrapolated from studies enrolling mixed cancer populations; (iii) their results were exclusively focused on the application of BIA for detecting lymphedema; and (iv) data derived from case reports, audits, surveys, or conference abstracts. 

### 2.3. Data Extraction

For each selected study, the following data were systematically extracted: (a) first author name and year of publication, country of origin; (b) study design and aims of the study; (c) sample size; (d) inclusion and exclusion criteria of participants; (e) state of cancer treatment (before, undergoing, or completed their therapy); (f) type of therapy (chemotherapy, hormone therapy, and/or radiotherapy); (g) information related to surgery; (h) study group; (i) age and anthropometric characteristics of subjects (body weight, stature, and BMI); (j) PhA values at 50 kHz and all impedance parameters; (k) FFM and fat mass (FM) with the relative equations used to estimate body composition, if available; (l) methods and instruments used for the analysis; (m) standardized conditions and position adopted for the measurements; and (n) indicators pertaining to the nutritional and functional status of patients.

### 2.4. Risk of Bias Assessment

The risk of bias assessment was evaluated using different tools developed jointly by methodologists from the National Heart, Lung, and Blood Institute and Research Triangle Institute International [29]. Tools evaluated potential flaws in study methodology, including sources of bias, confounding, study power, and other factors. A total of four options as a response: “yes”, “no”, “not reported (NR)”, or “not applicable (NA)”, can be appointed for each item. A judgment of “good” indicated a low risk of bias, “poor” indicated a significant risk of bias, and “fair” meant that the study was susceptible to some bias deemed not sufficient to invalidate its results.

## 3. Results

### 3.1. Selected Studies

**Figure 1:**
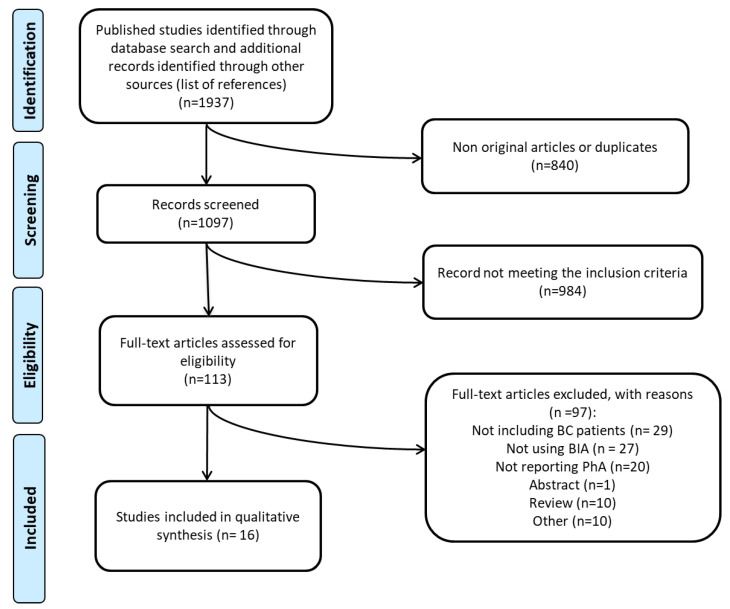
Flow diagram of the literature search process.

The initial literature search retrieved 1937 records (Figure 1). After removing duplicates, 1097 studies were screened for titles and abstracts, and 113 full texts were finally assessed for eligibility; 16 studies met the inclusion criteria and were included in the present systematic review.

The studies had been carried out in Brazil [30,31,32,33,34,35], Italy [36,37], Poland [38,39], Portugal [18,40], Germany [41], Mexico [42], Turkey [43], and USA [14] between 2008–2021, involving 877 (min 9 [42] and max 259 [14]) patients after diagnosis of BC and survivors [18,33,35,36,37,40], as defined by the authors themselves. To simplify, however, in the present review we will use the term “BC patients” to define patients during the whole trajectory of the disease [44].

Of the eleven selected studies with an observational design, five were cross-sectional studies [18,30,38,39,40], five were prospective cohort studies [31,32,33,34,35] and one was a retrospective cohort study [14]. The remaining five had an interventional study design, where one was a randomized controlled trial (RCT) [43] and four were clinical trials: three with a single group [36,37,42] and one using a non-randomized parallel group assignment (Table 1) [41].

The majority of studies enrolled both pre- and postmenopausal women, except two [35,37] including women in physiological or pharmacological-induced menopause. The mean age of participants varied between 44 [42] and 65 [35] years. At recruitment, the menopausal status of patients was specified by four studies only, and the percentage of post-menopausal women varied from 34% [31,32] to 59% [30] of the samples as a whole. As indicated in Table 1, the stage of disease was considered in 10 studies, with most of them including subjects with BC stage ≤ III.

Concerning BMI, patients were mostly overweight, although mean BMI showed a wide variability from 23.7 kg/m^2^ [41] up to 30.9 kg/m^2^ [30]; whereas waist circumference was reported by six studies only, with an average value of 87–101 cm [30,40]. Anthropometry was not shown by Gupta et al., 2008 [14].

BIA was performed before surgery in two studies [38,39], and after surgery (i.e., mastectomy or quadrantectomy) in seven [18,35,36,37,40,42,43], with no information available in the remaining seven [14,30,31,32,33,34,41]. Nine studies included patients before starting any oncologic treatments, such as adjuvant or neo-adjuvant therapy [30,31,32,33,34,37,38,39,42], one enrolled BC patients undergoing radiotherapy [41], one study included women during aromatase inhibitors (AIs) treatment [35]; whilst three studies recruited women after completing antineoplastic treatments [18,36,43]. Information about drugs therapy was missing in two studies [14,40].

### 3.2. BIA Methods, Instrument, and Measurement Conditions

The evaluation of PhA was one of the main objectives only in six papers [14,18,31,34,40,43]. In all selected studies PhA was measured at 50 kHz (in some cases also at other frequencies, not pertinent to this review).

As shown in Table 1, five single-frequency BIA [14,36,37,42,43], seven multi-frequency BIA [18,33,34,35,38,39,41] and three BIS analyzers [31,32,40] were used. With regard to measurement conditions, in all selected studies, except for two [41,43], BIA was performed in supine position. Six studies described bed rest before BIA, ranging from 5 [33] to 10 min [18,38,39,40], and fasting state was mentioned by four studies, varying from a 4 [42] until 8–12 h fast [18,33,40]. More than half of the studies did not report any information about fast and rest [14,30,31,32,34,35,36,37,41,43]. One study [30] did not provide information either on device or on patient’s position.

### 3.3. Risk of Bias

The risk of bias for the selected studies was reported in the Supplementary Materials. Based on the Quality Assessment Tool for Observational Cohort and Cross-Sectional Studies, the risk of bias was “good” in three studies [31,32,35], “fair” in five studies and “poor” in three papers (study population was not adequately described and potential confounding variables were not adjusted) (Table S1) [14,30,38]. None of the five interventional studies had a “good” risk of bias according to the specific tool adopt-ed, with two studies [36,42] rated as “poor” (participants were not representative of the population of interest and selection criteria were not prespecified) (Tables S2 and S3).

### 3.4. BIA-Derived PhA in BC Patients

**Figure 2:**
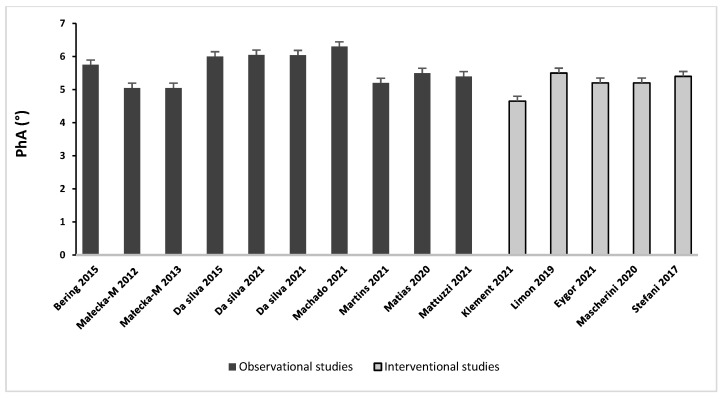
Baseline PhA values in patients with BC.

Results on PhA were presented according to study design and summarized in Table 2 and Table 3. Baseline values of PhA varied from 5.05 [38] to 6.30 degrees [33] in observational studies and from 4.49 [41] to 5.5 degrees [42] in interventional studies, as presented in Figure 2.

#### 3.4.1. Observational Studies

Eleven observational studies evaluating PhA in BC patients were reported in Table 2 as follows: four cross-sectional studies evaluated the differences between patients and controls [38,39] or within subgroups of BC patients [18,30]; three prospective studies investigated changes in PhA values after the end of chemotherapy (<10 months) [31,32,34], and two assessed the effect on PhA in the long term (≥2 years), considering the role of disease staging [33] and the impact of AIs treatment [35]. The association with survival was analyzed by a single study [14]. Then, 5 out 11 studies [18,32,38,39,40] assessed the link between PhA, nutritional status, and muscle strength.

##### Changes between Categories of BC Patients

One cross-sectional study [30] found that pre-therapy BC patients with metabolic syndrome (MeS) had significantly higher BMI and percentage of both FM and FFM compared to the ones without MeS, but PhA was similar between the two groups [30]. In another study [18], a small sample of BC patients (around six years from diagnosis) were subdivided into two subgroups (PhA below and above 5.6 degrees) according to a cutoff previously defined [14]. BC patients with higher PhA values exhibited a so called “better health status” and a lower ratio between ECW and TBW (Table S4), with no difference in body weight and body composition.

##### Changes after Cancer Treatments in the Short (<10 Months) and Long Term (>2 Years)

Three studies [31,32,34] have reported data about the effects of chemotherapy on PhA. Women with BC were prospectively evaluated before (T0), at the end (T1) and two months after (T2) the last chemotherapy. PhA was significantly reduced at T1 (−5%), but not at T2, compared to baseline value; whereas weight, and percentage of FM (as reported by the authors), increased over time [34]. Another prospective cohort study was carried out in a group of women with early BC (72% in neoadjuvant and 28% in adjuvant therapy) and results were published in two separate papers. PhA significantly decreased one month after completing treatments (−15% vs. baseline, mean follow-up 7 months), with 75.4% of women having low PhA according to the above-mentioned cut-off. Concurrently, ECW and the ECW/TBW ratio increased compared to baseline (Table S4), while no significant changes were reported for body weight, FFM, and FM.

In a recent study [33], PhA was evaluated at baseline (diagnosis) and 5 years after the last chemotherapy cycle (follow-up) in 35 women with BC subdivided according to their clinical tumoral staging: CS1 = initial (stage I-II) and CS2 = advanced (stage III-IV). At follow-up, there was a significant reduction in PhA values in the sample as a whole (−10%) as well as in both CS1 and CS2 groups (CS1: (T1) 5.7 ± 0.6 degrees vs. (T0) 6.4 ± 0.8 degrees, CS2: (T1) 5.5 ± 0.7 degrees vs. (T0) 6.1 ± 1.0 degrees). Moreover, women on CS1, but not on CS2, revealed a significant increase of %FM (+1.51%) and a decrease of %FFM (−1.51%) compared to their baseline values.

On the contrary, a reduced PhA value was observed in BC patients undergoing AIs at the beginning of the follow-up period (T0) compared to the intermediate (T1: 1 year) and the final follow-up (T2: 2 years); while no changes were observed in anthropometric and body composition data over time [35].

##### Association between PhA and Survival

A retrospective analysis evaluating the association of PhA with survival in 259 histologically confirmed BC patients [14], of whom 81 were newly diagnosed and 178 underwent prior treatment elsewhere, showed that PhA varied from 1.5 to 8.9 degrees with a median value of 5.6 degrees. The median survival was greater (*p* = 0.031) in patients with a PhA > 5.6 degrees (49.9 months; 95% CI: 35.6 to 77.8) than those with a PhA < 5.6 degrees (23.1 months; 95% CI 14.2 to 31.9).

##### Associations between PhA, Nutritional Status, and Muscle Strength

The associations of PhA with nutritional status and/or muscle strength were evaluated by four cross-sectional [18,38,39,40] and one prospective cohort studies [32].

In two papers by Małecka-Massalska et al. [38,39], the subjective global assessment (SGA) was used as tool for nutritional risk assessment, showing that both pre-operative BC patients and controls were well nourished with no difference in PhA.

The cross-sectional study by Matias et al., 2020 aimed to determine whether PhA can be used for predicting muscular strength (handgrip strength, HGS). In their sample of BC patients, PhA accounted for 22% of muscular strength variance.

Martins et al., 2021 found no differences in health-related physical fitness, assessed using 30 s sit-to-stand test, timed ‘up and go’ test, ball throw test (3 kg), and a 6 min walking test between the two subgroups of BC patients, divided according to a PhA value of 5.6 degrees (see above).

In the only prospective study, Da Silva et al., 2021 used the NRI for assessing the nutritional status and the HGS, along with the gait speed test (GS) for evaluating the physical fitness of patients. According to the nutritional risk index (NRI), the percentage of patients at moderate-severe risk rose from 31 to 66% one month after completing chemotherapy. Women at nutritional risk also had lower mean values of PhA at both time points (T0 and T1). Instead, a slight not significant decline of HGS (−1.4 kg) was observed after treatment, and a high prevalence of slowness before starting the chemotherapy (T0) and at the end of the study (T1). PhA is significantly correlated with variables related to nutritional status, physical function, and body composition for both time points; being mainly correlated with FFM, HGS, and NRI at T0, and with FFM, GS, and NRI at T1. Furthermore, multiple regression analysis showed that age and HGS were independent predictors of PhA at baseline, and of GS and FFM after treatment.

#### 3.4.2. Interventional Studies

A total of five interventional studies reporting data about PhA in BC patients were included in the present review (Table 3). Two studies evaluated the effect of a nutritional intervention during radiotherapy [41] and chemotherapy [42]; another two assessed the impact of an unsupervised exercise intervention during chemotherapy [37] and after the end of chemotherapy [36]; while the last one investigated the effect of Hatha yoga on PhA following BC treatment [43].

##### Changes after Nutritional Program

The study by Klement et al., 2020 [41] tested the effects of a dietary/physical activity intervention based on the “Paleolithic lifestyle” (PL) over 3–6 weeks in 11 BC patients undergoing radiotherapy, compared to patients given an unspecified standard diet (SD). A small but statistically significant increase of PhA was observed in the SD group, while in the PL group there was an opposite trend. The PL group experienced a higher decrease of body weight compared to the SD one, due to the reduction of FM with no variation in TBW, ECW, and ICW (Supplementary Table S4).

The second study [42] evaluated the effect of an individualized nutrition intervention program in nine BC patients during therapy (baseline and after 6 months) and observed a surprising increase of mean PhA (+2.1 degrees on median values), with eight patients achieving PhA above the cut-off proposed by Gupta et al. [14], and a decrease in body weight by 5.8 kg (Table 3).

##### Changes after Hatha Yoga and Exercise Programs

To investigate the effect of yoga on BC patients 31 women were included and randomized into two groups: yoga (*n* = 15) and controls (*n* = 16). The yoga group practiced Hatha yoga for 1 h, 2 days per week for 10 weeks, while patients in the control group were advised to do regular exercise. After 10 weeks, PhA did not change in both the yoga and controls in comparison with their baseline value, and no difference was found between the two groups. Data about weight and body composition were not reported.

The effects of an unsupervised exercise program (tailored according to the American College of Sports Medicine-ACSM guidelines) on PhA were reported by two interventional studies (no control group).

First, Stefani et al., 2017 [36] assessed the effect of a physical training program lasting 12 months in 28 BC patients. They recommended a combination of aerobic exercises, which consisted primarily of walking, and resistance training. PhA significantly increased at 6 (+6%) and 12 months (+9%) with a concomitant decrease in ECW, whereas there were no significant changes in body weight, percentage of FFM, and FM, as shown in Table 3.

**Figure 3:**
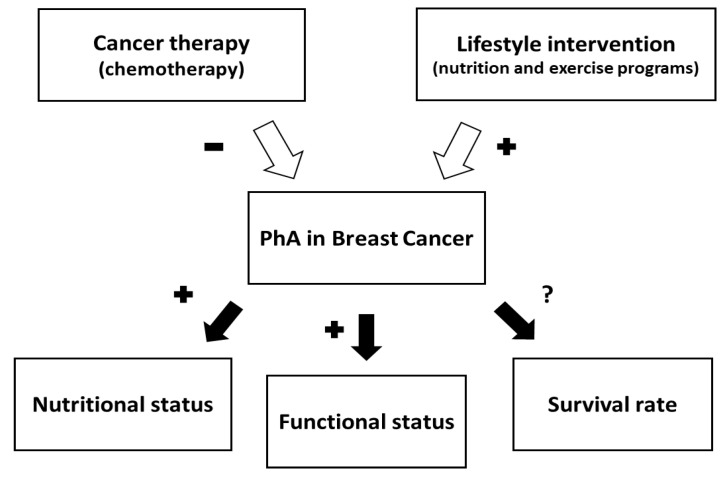
White arrows indicate that PhA is negatively affected by cancer therapy (−) and positively by lifestyle intervention (+). Black arrows indicate that PhA serves as a predictor of nutritional and functional status (+) in women with BC, while data are inconsistent about survival (?).

Similarly, Mascherini et al., 2020 [37] assessed the impact of an unsupervised exercise program characterized by both aerobic (150 min/week) and resistance training sessions (8 exercises involving the main muscle groups, performed for 3 sets with 10 repetitions twice per week) in BC patients over 6 months of hormone therapy and/or chemotherapy. At the end of the experimental period, PhA did not change substantially, whereas there was a progressive and significant decrease in weight (−3.2 ± 2.3 kg), FFM (−1.0 ± 2.9 kg) and FM (−2.4 ± 3.4 kg). Finally, the overall effect on PhA, and its use, in BC patients is summarized in Figure 3.

## 4. Discussion

The present systematic review set out to investigate available data concerning PhA in women with BC. We found a great variability and heterogeneity of PhA values across the selected studies, making it difficult to provide conclusive evidence. Available data suggested that PhA decreased significantly in BC patients after chemotherapy compared to baseline. The effects of oncologic therapies were evident in the short term, and possibly in the long term as well; indeed, it is likely that nutritional and/or exercise programs could prevent PhA reduction, and weight gain, during and after treatments.

Although cancer patients may experience detrimental changes in weight and adiposity during the course of the disease [3,45], each cancer type has its own unique characteristics with regards to body composition [46]. In BC patients an increase in percentage body fat and a decrease in lean mass are often described [47,48,49]; chemotherapy has been identified as one of the main contributors to weight gain over the entire trajectory of disease [5,10] and may induce alterations in TBW, FM, and FFM, even if no significant weight gain occurs [5,10,50]. In addition, recent studies have suggested that obesity is associated with poor outcomes and higher mortality in BC [4,10], regardless of when BMI is ascertained (i.e., before diagnosis, during or after primary treatment) [9]. The harmful effect of excess adipose tissue may be related to mechanisms such as increased estrogen production in adipose tissue, elevated insulin-like growth factor levels and altered synthesis of adipokines and cytokines, development of a chronic subclinical inflammation, etc. [51,52].

In the clinical evaluation of body composition, the assessment of raw BIA variables has gained increasing attention in order to obtain additional information on the inherent characteristics of FFM with low values of PhA indicating reduced BCM, altered cellular integrity, and high ECW/ICW ratio [11,26]. In the present systematic review, we intended to explore the available evidence on PhA in women with BC, with respect to differences vs. controls, variations due to cancer treatments and lifestyle interventions, and evaluation of nutritional status and survival.

A high prevalence of malnutrition was observed in certain types of cancers (for instance, head and neck, upper gastrointestinal, and lung), even before starting any oncologic treatments [53,54,55] and may be associated with an increase in the ECW/ICW ratio and a decrease in BCM, with consequent low PhA values [11,22,56,57]. So far, few and partial data are available in BC: two cross-sectional studies (the same small sample of patients) showed that PhA did not differ between preoperative BC patients and controls [38,39], with no malnutrition in both groups based on SGA; this is in line with the notion that in this type of cancer the prevalence of malnourished patients is low [58].

Chemotherapy may interfere with the patient’s lifestyle and nutritional status because of its secondary effects such as nausea, vomit, stypsis, diarrhea, anorexia, fatigue, asthenia, and peripheral neuropathy [42,59]. As a matter of fact, the detrimental impact of oncologic therapy on PhA was described by various studies [58,60,61,62]; for instance, radiotherapy at head and neck/upper abdomen/thorax was associated with significant weight loss and decrease in PhA when compared to other sites [58], while chemotherapy in head and neck cancer and advanced colorectal patients impaired PhA values with a concomitant increase in the prevalence of malnourished/cachectic patients [61]. So far, preliminary (but suggestive) findings are available for BC (five papers): PhA significantly declined (by 5–15%) after 7 months of adjuvant chemotherapy [31,32,34], while changes in weight or body composition were negligible. PhA was also reduced (~10%) 5 years after the last chemotherapy, but not following 2 years of AIs treatment [35], with no or minimal variations of weight [33] or BMI [35] compared to baseline. Changes in body composition (increased FM) were found only in the former study [33], which also reported a decrease in FFM values in women on early clinical tumoral staging. According to those preliminary findings, PhA may reveal some changes in FFM composition due to the complex alterations induced by side-effects of cancer treatments, which might not be revealed by weight changes.

In literature, it has been reported that lifestyle modifications (diet and exercise) may be effective in counterbalancing adverse changes of body composition in cancer patients [18,59,63]. In line with this idea, limited data suggest that PhA did not impair when tailored (quite different) nutritional interventions were carried out in BC patients during therapy, thus highlighting the importance of adequate nutritional programs [63]. Similar results concerning PhA have been reported in other studies as well, for instance in mixed cancer patients undergoing chemotherapy [64]. The increase in physical activity [65] is another feasible lifestyle modification with potential favorable effects; these latter, however, may depend on the type of exercise (practicing yoga seemed to be unsuccessful in BC patients) [43]. Actually, an unsupervised exercise program might be helpful for preventing PhA reduction during oncologic treatment in women with BC [37], and even more in the follow-up since PhA was found significantly increased and FM decreased [36]. These findings are in line with previous studies, for instance those carried out with patients undergoing allogeneic hematopoietic cell transplantation and post-colorectal cancer surgery [66,67].

Previous evidence has suggested that PhA is a valid index of nutritional status in cancer patients [8,11,21,26,44,59,68]. Some relationships between PhA and nutritional status have been found in different types of cancers (pancreatic, head and neck, colorectal, and mixed advanced tumors), with well-nourished patients showing a higher PhA compared to the malnourished ones [14,21,69]. Concerning the present review, PhA has also been found to be related to nutritional status in BC patients, showing lower values in women with an elevated nutritional risk [31,32], while it did not vary between well-nourished preoperative BC patients compared to controls [38,39]. PhA has been also linked with muscle strength in the oncologic setting [11,70,71]. A direct correlation between PhA and HGS was found in women with BC, regardless of age and level of physical activity [40]. In addition, during treatment women with lower PhA values showed a higher prevalence of malnutrition and a slight decline of muscle strength [31,32]. Similar findings were reported in mixed cancer populations, with patients with low PhA exhibiting decreased muscle strength compared with the ones with normal PhA [70,72].

Last, but not least, findings from recent meta-analyses suggested that PhA may be an important prognostic factor of survival in cancer patients [12,21]. A median PhA value of 5.6 degrees was originally identified in BC patients [14], with lower values associated with poor survival. Afterwards, few studies utilized that cutoff with inconsistent results: PhA values <5.6 degrees were found in women displaying a worsening overall “health status” [18], or at the end of chemotherapy [31,32], but they were also reported in apparently well-nourished pre- and post-surgery BC patients [40], and in healthy controls [38,39]. Overall, no appropriate statistical analysis (for instance, ROC curve) has so far been performed to identify a prognostic cutoff in BC patients, and there is no reason to accept the one proposed by Gupta et al., 2008 [14] as a valid reference value in nutritional studies because of drawbacks, relative to study design, population type, and methods for generating cutoff.

### Strength and Limitation

To the best of our knowledge, this is the first systematic review assessing BIA-derived PhA on BC patients. The strength of the present study was to provide a qualitative analysis about the use of PhA in this specific population, emphasizing its utility to identify and monitor changes in body composition over the whole trajectory of the disease.

Some limitations, however, need to be acknowledged. First, only a few papers have evaluated PhA as one of the main outcomes of the study. Secondly, the high heterogeneity of available data (menopausal status, tumor stage, cancer therapy, surgery, and follow-up time), the observational design of many of the included studies, the small sample size, and the poor quality of studies did not allow the provision of conclusive evidence. Finally, the use of different types of devices (single-, multi-frequency, and BIS), and the lack of detailed description of measurement conditions, might make the comparison more difficult and less coherent between different studies, affecting the results.

## 5. Conclusions

Current findings suggest that BIA-derived PhA significantly decreased after chemotherapy with variations that might occur during the whole trajectory of the BC disease. PhA emerged as a reliable predictor of nutritional and functional status in BC patients, while its association with survival is still unclear in this specific population. Currently, conclusive evidence is still lacking but in light of some promising findings, further studies, with strong quality design, are needed to focus on the clinical relevance of PhA in BC patients, evaluating its association with disease outcomes and survival, and to explore its usefulness in clinical practice.

## Figures and Tables

**Table 1:** Characteristics of the included studies.

Author, Year [Ref.]	Design	Study Population	N	Study Groups	Age (Years)	BMI (kg/m^2^)	BIA Methods/ Instrument	Measurements/Position
Observational Studies								
Bering et al., 2015 [30]	Cross-sectional	Pre/postmenopausal BC women; stage I–IV; age range: 31–79 y	64	MetS (*n* = 28) No-MetS (*n* = 36)	53.2 ± 11.6 ^‡^	30.9 ± 5.5 * 25.3 ± 4.3	NR	Fast and rest not specified
Małecka-Massalska et al., 2012 [38]	Cross-sectional	Pre/postmenopausal BC women; age range: 31–82 y; pre-surgery	68	BC (*n* = 34) C (*n* = 34)	53.88 ± 10.84 53.79 ± 10.18	26.97 ± 3.99 27.27 ± 7.66	Multifrequency ImpediMed SFB7 BioImp v1.5	10 min rest supine position
Małecka-Massalska et al., 2013 [39]	Cross-sectional	Pre/postmenopausal BC women; age range: 31–82 y; pre-surgery	68	BC (*n* = 34) C (*n* = 34)	53.88 ± 10.84 53.79 ± 10.18	26.0 ± 3.99 * 29.61 ± 7.66	Multifrequency ImpediMed SFB7 BioImp v1.5	10 min rest supine position
Martins et al., 2021 [18]	Cross-sectional	Pre/postmenopausal BC survivors; stage I–IIIA; age range: 30–69 y; post-surgery	25	G 1 (*n* = 13) G 2 (*n* = 12)	50.5 ± 8.6 51.1 ± 8.9	25.5 ± 3.9 27.3 ± 5.6	Multifrequency Biospace Co InBody S10	8 h fast and 10 min rest; supine position
Matias et al., 2020 [40]	Cross-sectional	Post-surgery BC survivors	41		54.6 ± 9.2	26.6 ± 4.6	Multifrequency BIS 4200B	Overnight fast; 10 min rest; supine position
Gupta et al., 2008 [14]	Retrospective cohort study	Pre/postmenopausal BC women; stage I–IV	259		49 (25–74) °	NR	Single frequency BIA-101Q: RJL Systems	NR Supine position
da Silva et al., 2015 [34]	Prospective cohort study	Pre/postmenopausal BC women; stage I–-II	25	T0 T1 (7 mo) T2 (9 mo)	46 (29–70)	25.0 ± 4.0 26.0 ± 5.0 * 26.0 ± 4.0 *	Multifrequency Biodynamics 450	Fast and rest not specified
da Silva et al., 2021 [31]	Prospective cohort study	Pre/postmenopausal BC women; stage I–III	61	T0 T1 (7 mo)	46.4 (26–64)	28.54 ± 5.46 28.95 ± 4.37	Multifrequency BIS-BCM Fresenius Medical care	Fast and rest not specified; supine position
da Silva et al., 2021 [32]	Prospective cohort study	Pre/postmenopausal BC women; stage I–III	61	T0 T1 (7 mo)	46.4 (26–64)	28.53 ± 5.45 29.23 ± 5.47	Multifrequency BIS-BCM Fresenius Medical care	Fast and rest not specified; supine position
Machado et al., 2021 [33]	Prospective cohort study	Pre/postmenopausal BC women, stage I–IV; aged ≥ 20 y	35	T0 T1 (5 y)	50.6 ± 11.4 55.5 ± 9.7	27.8 ± 4.4 28.5 ± 4.2	Multifrequency Biodynamics 450	12 h fast, 5 min rest; supine position
Mazzutti et al., 2021 [35]	Prospective cohort study	Postmenopausal BC survivors; stage I–III; post-surgery	38	T0 T1 (12 mo) T2 (24 mo)	65 (58.5–69.5) ^‡^ °	28.5 ± 1.10 29.2 ± 0.93 29.4 ± 1.12	Multifrequency Biodynamics 450	Fast and rest not specified; supine position
Interventional studies								
Klement et al., 2020 [41]	Clinical Trial	Pre/postmenopausal BC women	22	PL (*n* = 11) T0 T1 SD (*n* = 11) T0 T1	58 (37–72) ° 58 (35–67) °	24.2 (19.9–30.0) ° NR 23.7 (18.8–28.0) ° NR	Multifrequency Seca514–515 BC analyzers	Fast and rest not specified; stand position
Limon-Miro et al., 2019 [42]	Clinical trial	BC women; stage I-IIB post- surgery	9	T0 T1 (6 mo)	44 ± 12	30.7 (IQR 7–11) 29.5 (IQR 7–9)	Single frequency Impedimed Limited DF50 (BIVA)	4 h fast; rest and position not specified
Eyigör et al., 2021 [43]	RCT	BC women; age range: 18–70 y; post-surgery	31	Y (*n* = 15) T0 T1 (10 weeks) C (*n* = 16) T0 T1 (10 weeks)	51.40 ± 10.6 50.7 ± 7.6	26.0 ± 4.9 25.7 ± 4.5 25.6 ± 3.7 24.7 ± 3.5	Tanita-305 body-fat analyzer	Fast and rest not specified; stand position
Mascherini et al., 2020 [37]	Clinical Trial	Postmenopausal BC women; stage < IIIC; age range: 21–65 y; post-surgery	42	T0 T1 (6 mo)	52.0 ± 10.1	27.3 ± 4.2 26.1 ± 3.9 *	Single frequency BIA 101 Sport edition, Akern	Fast and rest not specified; supine position
Stefani et al., 2017 [36]	Clinical Trial	BC survivors; post-surgery and radiotherapy	28	T0 T1 (6 mo) T2 (12 mo)	59 ± 9	26.7 ± 5.4 26.6 ± 5.6 26.8 ± 5.8	Single frequency BIA 101, Akern	Fast and rest not specified; supine position

Data are expressed as mean ± standard deviation unless otherwise specified. BMI = body mass index; BC = Breast cancer; BIA = bioimpedance analysis; BIS = Bioimpedance spectroscopy; BCM = body composition monitor; C = control group; G 1 (phase angle ≤ 5.6 degrees); G 2 (phase angle ≥ 5.6 degrees); IQR = interquartile range; MetS = metabolic syndrome; mo = months; NR = Not reported; PL = Paleolithic lifestyle; SD = Standard diet; T0 = baseline; T1 and T2 = time follow-up; y = year; Y = yoga group. ^‡^ Age is referred to the whole sample (*n* = 78 [30]) and (*n* = 89 [35]); ° Data are expressed as median and range [14,41] or 25–75° percentiles [35]; * *p* < 0.05 between groups and vs. T0.

**Table 2:** Weight, phase angle and body composition variables extracted by observational studies.

Author, Year, Ref.	Study Design	Stage of Care	N	Study Group	Weight (kg)	PhA (Degrees)	FFM (kg or %)	FM (kg or %)	Major Findings on PhA
Bering et al., 2015 [30]	Cross- sectional	Pre-chemotherapy and radiotherapy	62	MetS (*n* = 28) No-MetS (*n* = 36)	NR	5.7 ± 0.8 5.8 ± 1.0	(%): 57.7 ± 6.2 (%): 63.8 ± 7.9 *	(%): 42.2 ± 6.2 (%): 35.4 ± 6.5 *	PhA was similar between the two BC groups
Małecka-Massalska et al., 2012 [38]	Cross- sectional	Preoperative	68	BC (*n* = 34) C (*n* = 34)	69.04 ± 12.56 70.07 ± 23.6	5.05 ± 0.66 5.22 ± 0.64	NR	NR	PhA was similar between BC and C
Małecka-Massalska et al., 2013 [39]	Cross- sectional	Preoperative	68	BC (*n* = 34) C (*n* = 34)	67.94 ± 12.56 79.07 ± 23.60 *	5.05 ± 0.12 5.25 ± 0.11	43.3 ± 1.1 50.5 ± 1.3 *	26 ± 3.9 29.6 ± 7.6 *	PhA was similar between BC and C
Martins et al., 2021 [18]	Cross- sectional	Completed therapy	25	G1 (*n* = 13) G2 (*n* = 12)	66.8 ± 10.1 67.5 ± 14.8	5.2 ± 0.26 5.9 ± 0.3 *	43.2 ± 4.9 43.2 ± 7.9	23.1 ± 8.9 25.5 ± 10.5	Better health status in G2 compared to G1
Matias et al., 2020 [40]	Cross- sectional	NR	41		68.0 ± 11.7	5.5 ± 0.7	NR	NR	PhA can predict muscular strength in BC survivors
Gupta et al., 2008 [14]	Retrospective cohort study	NR	259		NR	5.6 (1.5–8.9) ‡	NR	NR	PhA seemed to be a strong predictor of survival in BC.
da Silva et al., 2015 [34]	Prospective cohort study	Pre/post adjuvant chemotherapy	25	T0 T1 (7 mo) T2 (9 mo)	64 ± 13 66 ± 13 * 67 ± 13 *	6.0 ± 0.6 5.7 ± 0.6 * 5.9 ± 1.4	NR	(%): 29.7 ± 6.1 (%): 29.5 ± 6.5 * (%): 30.7 ± 5.4 ^§^	PhA significantly decreased (−5%) after treatments
da Silva et al., 2021 [31]	Prospective cohort study	Pre-/post (neo) adjuvant chemotherapy	61	T0 T1 (7 mo)	71.7 ± 12.6 73.5 ± 12.6	6.05 ± 0.75 5.16 ± 0.77 *	34 ± 7.1 32.5 ± 5.6	28.82 ± 9.09 28.78 ± 8.94	PhA significantly decreased (−15%) after treatments
da Silva et al., 2021 [32]	Prospective cohort study	Pre-/post (neo) adjuvant chemotherapy	61	T0 T1(7 mo)	71.7 ± 12.6 72.1 ± 12.4	6.04 ± 0.76 5.18 ± 0.76 *	34 ± 7.1 33.8 ± 8.40	28.82 ± 9.09 28.86 ± 10.04	PhA significantly decreased (−15%) after treatments
Machado et al., 2021 [33]	Prospective cohort study	Pre/post treatments	35	T0 T1 (5 y)	67.4 ± 11.2 71.0 ± 12.0	6.3 ± 0.9 5.7 ± 0.6 *	43.5 ± 3.3 44.7 ± 1.7	(%): 35.4 ± 4.9 (%): 37.0 ± 2.5 *	PhA significantly decreased (−10%) after follow-up
Mazzutti et al., 2021 [35]	Prospective cohort study	During AIs treatments	38	T0 T1 (12 mo) T2 (24 mo)	NR	5.4 ± 0.20 6.2 ± 0.11 * 6.1 ± 0.15 *	42.3 ± 1.08 43.3 ± 0.87 43.0 ± 1.15	(%): 40.5 ± 1.25 (%): 39.3 ± 1.02 (%): 39.7 ± 1.34	PhA was lower at T0 compared to T1 and T2

Data are expressed as mean and SD unless otherwise specified. AIs = Aromatase Inhibitors; BC = breast cancer; C = Controls; FFM = Fat-Free mass; FM = Fat Mass; kg = kilogram; MetS = Metabolic Syndrome; Group 1 (phase angle ≤ 5.6°); Group 2 (phase angle ≥ 5.6°); mo = months; NR = Not reported; PhA = phase angle; T0 = baseline; T1 and T2 = time follow-up; y = years. FFM data were converted from % to kg in all studies, unless otherwise specified. ^‡^ Data are expressed as median and range; * *p* < 0.05 between groups or vs. T0; ^§^ *p* < 0.05 vs. T1 and T0.

**Table 3:** Weight, phase angle and body composition variables extracted by the interventional studies.

Author, Year, Ref.	Study Design	Stage of Care	N	Study Group	Weight (kg)	PhA (Degrees)	FFM (kg or %)	FM (kg or %)	Major Findings
Klement et al., 2020 [41]	Clinical trial	Undergoing radiotherapy	22	PL (*n* = 11) T0 T1 (39 days) SD (*n* = 11) T0 T1 (33 days)	62.5 (54.1–88.4) − 0.4/week * 61.3 (48.1–75.2) NR	4.81 (4.04–5.28) − 0.02/week 4.49 (3.87–5.37) 0.03/week *	40.1 (34.2–51.5) 0.04/week 38.7 (31.5–48.7) −0.13/week *	22.4 (16.5–37.7) − 0.34/week * 21.6 (15.9–31.7) 0.14/week	PhA values show an opposite trend between diets
Limon-Miro et al., 2019 [42]	Clinical trial	Pre-/post adjuvant chemo- and/or radiotherapy	9	T0 T1 (6 mo)	79.2 (IQR 10–27) 73.4 (IQR 13–22) *	5.5 (IQR 3–10) 7.6 (IQR 4–10) *	NR	NR	PhA significantly improved after nutrition intervention (+38%)
Mascherini et al., 2020 [37]	Clinical trial	Pre/post adjuvant hormone and/or chemotherapy	42	T0 T1 (6 mo)	71.9 ± 10.8 68.7 ± 10.1 *	5.2 ± 0.7 5.3 ± 0.7	46.7 ± 4.7 45.7 ± 4.4 *	25 ± 8.1 22.6 ± 7.2 *	PhA remained stable before and after 6 mo from starting therapy (+2%)
Stefani et al., 2017 [36]	Clinical Trial	Completed therapy	28	T0 T1 (6 mo) T2 (12 mo)	70.2 ± 9.9 69.9 ± 14.9 70.5 ± 15.8	5.4 ± 0.7 5.7 ± 0.8 * 5.9 ± 0.7 *	45.9 ± 5.9 ^•^ 45.8 ± 5.9 ^•^ 46.7 ± 7.2 ^•^	(%): 34.6 ± 8.3 (%): 34.4 ± 8.5 (%): 33.7 ± 10.3	PhA significantly improved after exercise program (+9%)
Eyigör et al., 2021 [43]	RCT	Completed therapy	31	Y (*n* = 15) T0 T1 (10 weeks) C (*n* = 16) T0 T1 (10 weeks)	NR	5.2 ± 0.7 5.2 ± 0.5 5.2 ± 0.4 5.2 ± 0.5	NR	NR	PhA was not affected by Hatha yoga exercises. No difference was found between the two groups

Data are expressed as mean and SD unless otherwise specified. C = Controls; mo = months; IQR = Interquartile range; PhA = Phase angle; FFM = Fat-Free mass; FM = Fat Mass; kg = kilogram; T0 = baseline; T1 and T2 = time follow-up; NR = Not reported; PL = Paleolithic Lifestyle; RCT = randomized controlled trial; SD = Standard Diet; Y = Yoga Group. The study by Klement et al. [41] expressed data as median and range at T0 and as difference from baseline in kg/week at T1; • FFM data are converted from % to kg; * *p* < 0.05 vs. T0.

## Data Availability

The data presented in this study are available in this article and Supplementary Material.

## Supplementary Materials

The following supporting information can be downloaded at: https://www.mdpi.com/article/10.3390/cancers14082002/s1, Table S1: Quality Assessment Tool for Observational Cohort and Cross-Sectional Studies; Table S2: Quality Assessment Tool for Before-After (Pre-Post) Studies with No Control Group; Table S3: Quality Assessment Tool of Controlled Intervention Studies; Table S4: Total body water, intra- and extracellular water assessed by BIA in patients with breast cancer.
