# Supplementary material for: Bioelectrical Phase Angle in Patients with Breast Cancer: A Systematic Review
Source: Cancers (Basel). 2022 Apr 15;14(8):2002. doi: 10.3390/cancers14082002 (PMC9025027; doi:10.3390/cancers14082002)
Supplement: Supplementary file 1 [file cancers-14-02002-s001.zip › cancers-1670392-supplementary.pdf]

Table S1. Quality Assessment Tool for Observational Cohort and Cross-Sectional Studies.

Criteria	Bering et al. 2015 [30]	da Silva et al. 2015 [34]	da Silva et al. 2021[a] [31]	da Silva et al. 2021[b] [32]	Gupta et al. 2008 [14]	Machado et al.2021[33]	Małecka-Massalska et al. 2012 [38]	Małecka-Massalska et al. 2013 [39]	Martins et al. 2021[18]	Matias et al. 2020 [40]	Mattuzzi et al. 2021 [35]
1. Was the research question or objective in this paper clearly stated?	Yes	Yes	Yes	Yes	Yes	Yes	Yes	Yes	Yes	Yes	Yes
2. Was the study population clearly specified and defined?	No	No	Yes	Yes	No	Yes	No	Yes	No	No	Yes
3. Was the participation rate of eligible persons at least 50%?	No	Yes	Yes	Yes	No	No	No	No	No	No	Yes
4. Were all the subjects selected or recruited from the same or similar populations (including the same time period)? Were inclusion and exclusion criteria for being in the study prespecified and applied uniformly to all participants?	Yes	Yes	Yes	Yes	No	Yes	No	Yes	Yes	Yes	Yes
5. Was a sample size justification, power description, or variance and effect estimates provided?	No	No	No	Yes	No	No	No	No	No	Yes	Yes
6. For the analyses in this paper, were the exposure(s) of interest measured prior to the outcome(s) being measured?	No	Yes	Yes	Yes	No	Yes	No	No	No	No	Yes

7. Was the timeframe sufficient so that one could reasonably expect to see an association between exposure and outcome if it existed?	No	Yes	No	Yes	Yes	Yes	No	No	No	No	Yes
8. For exposures that can vary in amount or level, did the study examine different levels of the exposure as related to the outcome (e.g., categories of exposure, or exposure measured as continuous variable)?	NA	NA	NA	NA	NA	NA	NA	NA	NA	NA	NA
9. Were the exposure measures (independent variables) clearly defined, valid, reliable, and implemented consistently across all study participants?	Yes	Yes	Yes	Yes	Yes	Yes	Yes	Yes	Yes	Yes	Yes
10. Was the exposure(s) assessed more than once over time?	No	Yes	Yes	Yes	No	Yes	No	No	No	No	Yes
11. Were the outcome measures (dependent variables) clearly defined, valid, reliable, and implemented consistently across all study participants?	Yes	Yes	Yes	Yes	Yes	Yes	Yes	Yes	Yes	Yes	Yes
12. Were the outcome assessors blinded to the exposure status of participants?	NA	NA	NA	NA	NA	NA	NA	NA	NA	NA	NA
13. Was loss to follow-up after baseline 20% or less?	NA	Yes	Yes	Yes	NA	No	NA	NA	NA	NA	Yes
14. Were key potential confounding variables measured and adjusted statistically for their impact on the	No	Yes	Yes	Yes	Yes	No	No	No	Yes	Yes	Yes

relationship between exposure(s) and outcome(s)?											
**Quality Rating (Good, Fair, or Poor)**	**Poor**	**Fair**	**Good**	**Good**	**Poor**	**Fair**	**Poor**	**Fair**	**Fair**	**Fair**	**Good**
**Comments (If POOR, please state why):**	Less than 50% of eligible people participated and they were not clearly specified				Less than 50% of eligible people participated and they were not clearly specified.		Less than 50% of eligible people participated and they were not clearly specified.				

Rater #1 Initials: D.M.

Rater #2 Initials: I.C.

NA, not applicable; NR, not reported and CD, cannot determine. The Quality Rating of each study was rated as Good, Fair or Poor.

Table S2. Quality Assessment Tool for Before-After (Pre-Post) Studies with No Control Group.

**Criteria**	**Klement et al. 2020 [41]**	**Limon-Miro et al. 2019 [42]**	**Mascherini et al. 2020 [37]**	**Stefani et al. 2017 [36]**
1. Was the study question or objective clearly stated?	Yes	Yes	Yes	Yes
2. Were eligibility/selection criteria for the study population prespecified and clearly described?	Yes	Yes	Yes	No
3. Were the participants in the study representative of those who would be eligible for the test/service/intervention in the general or clinical population of interest?	No	No	Yes	No
4. Were all eligible participants that met the prespecified entry criteria enrolled?	Yes	No	No	No
5. Was the sample size sufficiently large to provide confidence in the findings?	No	Yes	Yes	No
6. Was the test/service/intervention clearly described and delivered consistently across the study population?	Yes	No	Yes	Yes
7. Were the outcome measures prespecified, clearly defined, valid, reliable, and assessed consistently across all study participants?	Yes	Yes	Yes	Yes
8. Were the people assessing the outcomes blinded to the participants' exposures/interventions?	No	No	No	No
9. Was the loss to follow-up after baseline 20% or less? Were those lost to follow-up accounted for in the analysis?	Yes	No	No	No
10. Did the statistical methods examine changes in outcome measures from before to after the intervention? Were statistical tests done that provided p values for the pre-to-post changes?	Yes	Yes	Yes	Yes
11. Were outcome measures of interest taken multiple times before the intervention and multiple times after the intervention (i.e., did they use an interrupted time-series design)?	NR	NR	NR	NR
12. If the intervention was conducted at a group level (e.g., a whole hospital, a community, etc.) did the statistical analysis take into account the use of individual-level data to determine effects at the group level?	Yes	Yes	Yes	Yes
**Quality Rating (Good, Fair, or Poor)**	**Fair**	**Poor**	**Fair**	**Poor**

Comments (If POOR, please state why):		Participants were not representative of the population of interest.		Low n. of participants and selection criteria were not prespecified.

Rater #1 Initials: D.M.

Rater #2 Initials: I.C.

NA, not applicable; NR, not reported and CD, cannot determine. The Quality Rating of each study was rated as Good, Fair or Poor.

Table S3. Quality Assessment Tool of Controlled Intervention Studies.

**Criteria**	**Eygor et al. 2021 [43]**
1. Was the study described as randomized, a randomized trial, a randomized clinical trial, or an RCT?	Yes
2. Was the method of randomization adequate (i.e., use of randomly generated assignment)?	Yes
3. Was the treatment allocation concealed (so that assignments could not be predicted)?	Yes
4. Were study participants and providers blinded to treatment group assignment?	No
5. Were the people assessing the outcomes blinded to the participants' group assignments?	Yes
6. Were the groups similar at baseline on important characteristics that could affect outcomes (e.g., demographics, risk factors, co-morbid conditions)?	Yes
7. Was the overall drop-out rate from the study at endpoint 20% or lower of the number allocated to treatment?	Yes
8. Was the differential drop-out rate (between treatment groups) at endpoint 15 percentage points or lower?	Yes
9. Was there high adherence to the intervention protocols for each treatment group?	NR
10. Were other interventions avoided or similar in the groups (e.g., similar background treatments)?	Yes
11. Were outcomes assessed using valid and reliable measures, implemented consistently across all study participants?	Yes
12. Did the authors report that the sample size was sufficiently large to be able to detect a difference in the main outcome between groups with at least 80% power?	No
13. Were outcomes reported or subgroups analyzed prespecified (i.e., identified before analyses were conducted)?	No
14. Were all randomized participants analyzed in the group to which they were originally assigned, i.e., did they use an intention-to-treat analysis?	Yes
**Quality Rating (Good, Fair, or Poor)**	**Fair**
**Comments (If POOR, please state why):**	

Rater #1 Initials: D.M.

Rater #2 Initials: I.C.

NA, not applicable; NR, not reported and CD, cannot determine. The Quality Rating of each study was rated as Good, Fair or Poor.

Table S4. Total body water, intra- and extracellular water assessed by BIA in patients with breast cancer

Author, Year ref	Study design	Stage of care	N	Study group	TBW (L)	ECW (L)	ICW (L)	ECW/TBW (L)
**Observational studies**								
Martins et al. 2021 [18]	Cross-sectional	Completed therapy	25	G 1 (n=13)	31.7 ± 3.5	12.2 ± 1.4	19.5 ± 2.1	0.40 ± 0.004
				G 2 (n=12)	31.7 ± 5.9	12.0 ± 2.3	19.7 ± 3.6	0.37 ± 0.004*
Matias et al. 2020 [40]	Cross-sectional	NR	41		NR	13.9±1.7	16.1 ± 3	NR
da Silva et al. 2021 [32]	Prospective cohort study	Pre-/post (neo) adjuvant chemotherapy	61	T0	31.9 ± 5.12	14.35 ± 2.20	17.54 ± 3.25	0.45 ± 0.02
				T1 (7 mo)	33.22 ± 6.10	16.00 ± 3.09 *	17.22 ± 3.33	0.48 ± 0.02**
**Interventional studies**								
Klement et al. 2020 [41]	Clinical trial	Undergoing radiotherapy	22	PL (n=11)				
				T0	30.1 (25.8–38.2)	13.8 (12.6–17.6)	16.5 (13.2–20.6)	0.46§
				T1 (39 days)	0.04 ± 0.05	0.08 ± 0.03 *	-0.03 ± 0.05	
				SD (n=11)				
				T0	29.9 (23.8–36.2)	13.9 (11.1–16.9)	15.9 (12.7–20.0)	0.47§

				T1 (33 days)	NR	NR	NR	
Mascherini et al. 2020 [37]	Clinical trial	Pre/post adjuvant hormone and/or chemotherapy	42	T0	35 ± 3.3	17.5 ± 1.9	17.5 ± 2.3	0.50§
				T1 (6 mo)	34.2 ± 3.3*	16.8 ± 1.9*	17.4 ± 2.2	0.49§
Stefani et al. 2017 [36]	Clinical Trial	Completed therapy	28	T0	49.2 ± 5.6	48.9 ± 3.9	51.1 ± 3.9	0.99§
				T1 (6 mo)	49.5 ± 6.0	47.6 ± 4.1*	52.4 ± 4.1*	0.96§
				T2 (12 mo)	50.0 ± 7.0	46.4 ± 3.1*	53.6 ± 3.1*	0.93§

Data are expressed as mean ± SD unless otherwise specified. G 1 (phase angle ≤5.6 degrees); G 2 (phase angle≥5.6 degrees); mo= months; PL= Paleolithic lifestyle; SD= Standard diet; T0= baseline; T1 and T2= time follow-up; TBW= total body water; ECW= extracellular water; ICW= intracellular water; NR= not reported. The study by Klement et al. [41] reported data as median and range at T0 and as difference from baseline in L/week at T1; *p<0.01; **p< 0.001; § data were calculated.
